# From Sound to Stability: Lessons Learned From the CRUSH Study on Hearing Loss Progression and Vestibular Phenotype in Usher Syndrome Type 2A

**DOI:** 10.1097/MAO.0000000000004851

**Published:** 2026-02-23

**Authors:** Dirk H. Wijn, Mirthe L.A. Fehrmann, Sybren M.M. Robijn, Hedwig M. Velde, Jeroen J. Smits, Erwin van Wijk, Andy J. Beynon, Froukje L.J. Cals, Carel B. Hoyng, Suzanne Yzer, Cris P. Lanting, Ronald J.E. Pennings

**Affiliations:** a Department of Otorhinolaryngology, Radboud University Medical Center, Nijmegen, the Netherlands; b Department of Clinical Genetics, Radboud University Medical Center, Nijmegen, the Netherlands; c Department of Ophthalmology, Radboud University Medical Center, Nijmegen, the Netherlands

**Keywords:** Natural history study, Sensorineural hearing loss, Usher syndrome, Vestibular testing

## Abstract

**Objective:**

This study aims to analyze the progression of hearing loss using prospectively collected data and describe the vestibular phenotype of Usher syndrome type 2A (USH*2a*).

**Study Design:**

Longitudinal, prospective, observational natural history study.

**Patients:**

Patients with USH2a and *USH2A*-associated non-syndromic retinitis pigmentosa (nsRP).

**Main Outcome Measures:**

Hearing loss progression was measured by pure tone audiometry (PTA) and phoneme scores. Vestibular function was assessed by velocity step tests (VSTs), video head impulse tests (vHITs), caloric reflex tests, and vestibular evoked myogenic potentials (VEMPs). Patient-reported symptoms were assessed by questionnaires (Usher Lifestyle Survey, SF-12, PHQ-9, DHI, and SSQ).

**Results:**

Thirty-three patients with USH2a and 2 patients with nsRP were included. PTA_0.5-4kHz_ thresholds showed a significant decrease of 2.3 dB (*P*=0.017) over 4 years. The progression of hearing loss was most pronounced in the mid-to-high frequencies. No significant differences were observed in phoneme scores as measured by the Speech Reception Threshold (SRT). The 2 patients with nsRP had normal hearing. Vestibular evaluation showed abnormal cervical VEMPs in 34% and abnormal ocular VEMPs in 75% of USH2a patients. VSTs, caloric reflex tests, and vHIT results were within normal limits in more than 90% of subjects. Patient questionnaires revealed no major balance problems as indicated by the DHI.

**Conclusions:**

This analysis provides robust evidence of measurable hearing loss progression in PTA_0.5-4kHz_ thresholds in USH2a patients beyond presbycusis. Speech understanding, as measured by SRT, remains relatively stable. Vestibular evaluation revealed no semicircular canal dysfunction, although VEMP results suggest a potential subclinical otolith organ impairment in USH2a patients.

## Introduction

Usher syndrome is an autosomal recessive genetic condition marked by sensorineural hearing loss (SNHL), progressive visual deterioration due to retinitis pigmentosa (RP), and variable vestibular dysfunction. It is the leading cause of deaf-blindness worldwide and is estimated to affect 4.4 to 6.2 people per 100,000.^1–3^ Usher syndrome is genetically heterogeneous, with 9 genes so far strongly linked to the condition and more potentially associated.^4^

**Figure 1:**
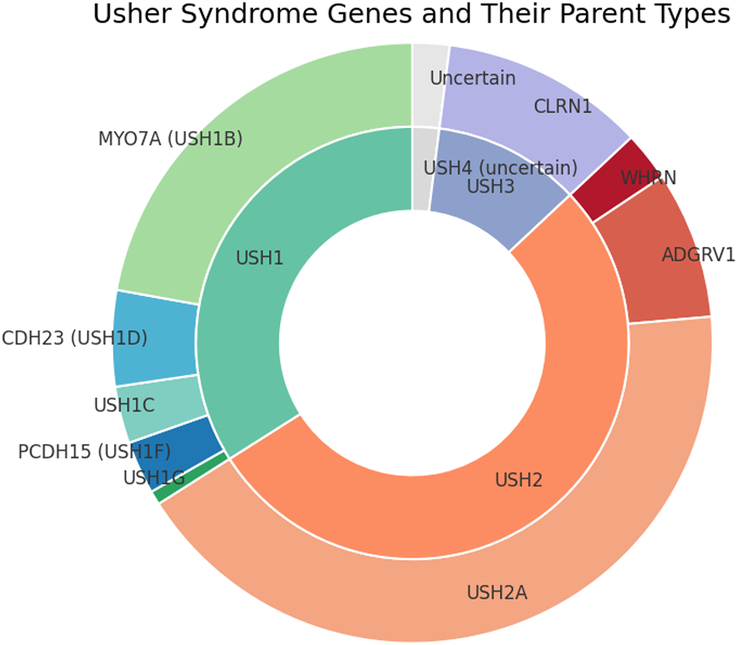
Diagram summarizing the Usher syndrome subtypes.

The disorder is classically divided into 3 types, each varying in severity, age of onset of hearing and vision loss, and whether vestibular dysfunction is present. Usher syndrome type II (USH2) is the most common type of Usher syndrome, accounting for 47% to 59% of all cases.^5,6^ It is associated with pathogenic variants in 3 genes: *USH2A*, *ADGRV1*, and *WHRN*, with variants in *USH2A* being the most prevalent. Pathogenic variants in *USH2A* are found in 57% to 79% of all USH2 cases.^7^ (Fig. 1) Mutations in the *USH2A* gene can also cause non-syndromic retinitis pigmentosa (nsRP): *USH2A*-associated nsRP.

USH2 is associated with mild to moderate congenital high-frequency sensorineural hearing impairment, particularly in the higher frequencies.^8–10^ While hearing loss is not typically associated with *USH2A*-related nsRP, a study by Hartel et al.^11^ found that almost half of the affected individuals showed signs of mild adult-onset hearing impairment beyond standard presbycusis. Early intervention with hearing aids is the standard strategy for managing USH2-related hearing loss, although some patients experience further decline, leading to difficulties with speech recognition. Cochlear implants (CIs) offer a viable solution in these cases, significantly enhancing speech perception and overall quality of life.^12,13^ USH2 is generally not associated with vestibular symptoms, but some studies have shown mild abnormalities in vestibular testing in a subset of patients.^14,15^

The onset of vision loss due to RP, a degenerative disease, typically occurs during puberty in USH2 patients. This inherited retinal disease is characterized by progressive loss of photoreceptors. The rod photoreceptors degenerate first, leading to night blindness and a progressive constriction of visual fields. Ultimately, there is also cone involvement, leading to loss of central vision and legal blindness. Most patients experience severe vision loss by the age of 40, although the rate of decline varies widely.^8^ Patients with *USH2A*-associated nsRP tend to experience slower visual deterioration than those with Usher syndrome type 2A (USH2a).^16^ Currently, RP in USH2 and nsRP patients is considered untreatable. However, several promising gene-targeted therapies, including antisense oligonucleotide-mediated splice modulation, CRISPR-Cas9 gene editing, and exon skipping, are in various stages of preclinical and clinical development, with a recently started phase 2b trial investigating a RNA therapy to promote *USH2A* exon 13 skipping.^17,18^

Understanding the natural progression of vision and hearing loss is essential to assess the effectiveness of developing gene-targeted therapies and to guide patient counseling. To address this need, the Characterizing Rate of Progression in USHer Syndrome (CRUSH) study was initiated in 2019. The main objective of this study is to map the natural course of visual and hearing deterioration in USH2a and *USH2A*-associated nsRP, providing essential insights for future genetic therapy studies. Secondary objectives are to improve counseling of patients with USH2a and *USH2A*-associated nsRP by providing a comprehensive audiological and vestibular phenotype and to identify additional etiological factors that may explain the variability in hearing impairment among individuals with USH2a.

## Methods

### Study design

The CRUSH study is a single-center, longitudinal, prospective natural history study on visual and hearing deterioration in patients with USH2a and *USH2A*-associated nsRP with a 4-year follow-up period. The study design is based on the RUSH2A study.^19^ The protocol is registered in ToetsingOnline (reference number NL67258.091.18) and the study was approved by the Medical Ethics Committee Oost-Nederland and adheres to the tenets of the Declaration of Helsinki. Informed consent was obtained from all participants before their participation in the study.

### Study population

Patients with USH2a or *USH2A*-associated nsRP were recruited from the Nijmegen Usher syndrome database for inclusion in the CRUSH study. Patients needed to have a clinical diagnosis of rod-cone degeneration, carry biallelic (likely) pathogenic variants in *USH2A*, have a Snellen equivalent of 20/80 or better, and be 16 years of age or older to be eligible for the study. *Appendix 1*, Supplemental Digital Content 1, http://links.lww.com/MAO/C365 provides a complete list of all inclusion and exclusion criteria. Only patients with a confirmed clinical and genetic diagnosis of USH2a or *USH2A*-associated nsRP were included.

### Study procedures and questionnaires

The CRUSH study collected extensive data on vision, hearing, and the vestibular system. This article discusses only the audiological and vestibular measurements.

All patients had a baseline visit at the beginning of the study. Demographic data and medical history were collected to assess risk factors associated with disease severity. Unaided hearing was assessed by pure tone audiometry (PTA), phoneme tests, distortion product otoacoustic emissions (DPOAEs), and digits-in-noise (DIN) tests. At the beginning of the study, patients also completed 5 questionnaires. Three questionnaires addressed their general well-being: the Short Form Health Survey 12 (SF-12), the Patient Health Questionnaire-9 (PHQ-9), and the Usher Lifestyle Survey. In addition, patients completed a questionnaire focused on hearing, the Speech, Spatial and Qualities of Hearing Scale (SSQ), and on balance, the Dizziness Handicap Inventory (DHI). These tests and questionnaires were repeated at the end of the study after 4 years, except for the DIN test. The vestibular examination was carried out in either the third or fourth year of the study. The vestibular system was assessed using velocity step tests (VSTs), video head impulse tests (vHITs), caloric reflex tests, and vestibular evoked myogenic potentials (VEMP) testing. *Appendix 2*, Supplemental Digital Content 2, http://links.lww.com/MAO/C366 provides an overview of the visit schedule and testing procedures.

### Statistical analysis

All data collected were anonymized and stored in CastorEDC for subsequent analysis. The PTA for each ear was calculated using thresholds at 0.5, 1, 2, and 4 kHz (PTA_0.5-4kHz_) and the mean PTA for both ears was used for analysis. The air conduction PTA_0.5-4kHz_ was used to categorize the degree of hearing loss according to the British Society of Audiology (BSA) classification.^20^

The Shapiro-Wilk test was used to assess the normality of the data. Sigmoid curve fitting was applied to the speech audiometry data to estimate SRTs. Linear mixed effects models were used to assess differences in PTA thresholds and SRTs between visits. Linear regression analysis was used to identify baseline predictors of mean PTA and to examine the relationship between hearing loss and age across frequencies, generating Age-Related Typical Audiograms (ARTA).^21^ The Kruskal-Wallis test was used to assess differences in mean PTA between subjects with truncating and nontruncating mutations and a Fisher exact test was used to compare VEMP measurements between these groups. Wilcoxon signed-rank tests were used to compare hearing threshold progression between visits across frequencies, both before and after adjustment for normal presbycusis based on the ISO 7029:2017 standard.^22^ A power analysis was performed using the observed PTA progression and variability to determine the sample size required to detect significant differences between groups for a potential placebo-controlled trial. A target power of 0.8 and a significance level of 0.05 (2-tailed) were set for the analysis, with results corrected for normal presbycusis progression based on ISO 7029:2017. Descriptive statistics were used to summarize the vestibular assessment data. All statistical analyses were performed using IBM SPSS Statistics (version 29.0) and R Studio (version 2024.12.0), with statistical significance set at a *P*-value of <0.05.

## Results

### Study population

A total of 41 patients were screened in the CRUSH study, all of whom underwent a baseline visit.

After this initial visit, 4 patients did not meet the inclusion criteria for follow-up due to a remaining kinetic visual field area of less than 7.5 degrees. During the 4-year follow-up, 2 patients withdrew from the study, leaving 35 patients for the final analysis. Of these, 33 had USH2a and 2 had *USH2A*-associated nsRP. One patient received a bilateral CI during the study and was therefore excluded from the analysis of audiological deterioration. The enrollment flowchart is shown in *Appendix 3*, Supplemental Digital Content 3, http://links.lww.com/MAO/C367.

Baseline characteristics are presented in Table 1, categorized by clinical diagnosis. The cohort consisted of 20 females and 15 males with a mean age of 36±10 years. All patients had biallelic (likely) pathogenic variants in *USH2A*. SNHL was diagnosed congenitally in most patients (77%), and at baseline, 40% had severe to profound SNHL according to the BSA classification. Of the patients with USH2a, 22 (67%) reported occasional or more frequent balance problems, whereas the 2 patients with nsRP did not report any balance problems. In the USH2a group, the mean PTA_0.5-4kHz_ was 66 dB HL (SD 14), whereas the 2 patients with nsRP had normal hearing (PTA_0.5-4kHz_ of 1 and 8 dB HL). Among the USH2a patients, the majority (91%) used a hearing aid at baseline, and none had a CI. *Appendix 4*, Supplemental Digital Content 4, http://links.lww.com/MAO/C369 provides an overview of the audiological characteristics categorized by diagnosis and *Appendix 5*, Supplemental Digital Content 5, http://links.lww.com/MAO/C370 lists the genotypes within this cohort.

**Table 1:** Key baseline characteristics by clinical diagnosis

Characteristic	Total Study Population (N=35)	USH2a (N=33)	nsRP (N=2)^a^
Gender, N (%)			
Female	20 (57)	19 (58)	1 (50)
Age at enrolment, y			
Mean (SD)	35.7 (10.3)	36.5 (10.1)	22 and 24
Age of onset visual symptoms, y			
Median (IQR)	16 (13.5-20)	16 (14-20)	12 and 15
[Min, Max]	[0.5, 47]	[0.5, 47]	
Age of onset hearing loss, N (%)			
Congenital	27 (77)	27 (82)	–
Prelingual	2 (6)	2 (6)	–
Postlingual	3 (9)	3 (9)	–
Unknown	1 (3)	1 (3)	–
No hearing loss	2 (6)	–	2 (100)
Severity of hearing loss, N (%)			
Normal (≤20 dB HL)	2 (6)	–	2 (100)
Mild (>20-≤40 dB HL)	1 (3)	1 (3)	–
Moderate (>40-≤70 dB HL)	18 (51)	18 (55)	–
Severe (>70-≤95 dB HL)	13 (37)	13 (39)	–
Profound (>95 dB HL)	1 (3%)	1 (3%)	–
Balance problems, N (%)			
Yes, regularly	4 (11)	4 (12)	–
Yes, sometimes	18 (51)	18 (55)	–
Unknown	1 (3)	1 (3)	–
No	12 (34)	10 (30)	2 (100%)
Age of onset balance problems, y			
Mean±SD	21.7 (16.8)	21.7 (16.8)	–

^a^ The 2 exact values of the 2 nsRP patients are given instead of the mean or median.

### Audiological findings

The estimated marginal mean PTA_0.5-4kHz_ of the USH2a patients increased from 66.1 dB at baseline to 68.5 dB at the end of the 4-year follow-up period. The contrast analysis showed a mean difference in PTA between visits of −2.3 dB with a CI of [−4.25 to −0.42] dB, corresponding to an annual threshold deterioration (ATD) of 0.6 dB per year. This effect is statistically significant (*P*=0.017), indicating an observable progression of hearing loss between visits. The progression is mainly found in the mid-to-high frequencies, with an ATD of 0 to 0.6 dB/year at 0.125 to 2 kHz and 1.0 to 1.6 dB/year at 4 to 8 kHz. A linear regression model assessed the association between mean PTA and baseline characteristics. Age was the only variable that showed a significant effect on mean PTA (*P*=0.015), with older age being associated with increased PTA. No significant associations were found for gender, history of ear infections, long-term antibiotic use, noise exposure, head injury, or meningitis. Furthermore, the effect of truncating and nontruncating variants was analyzed by forming 3 groups: individuals with 2 truncating variants, one truncating variant, and no truncating variants, with no statistically significant difference observed (*P*=0.21, Ɛ^2^=0.019).

**Figure 2:**
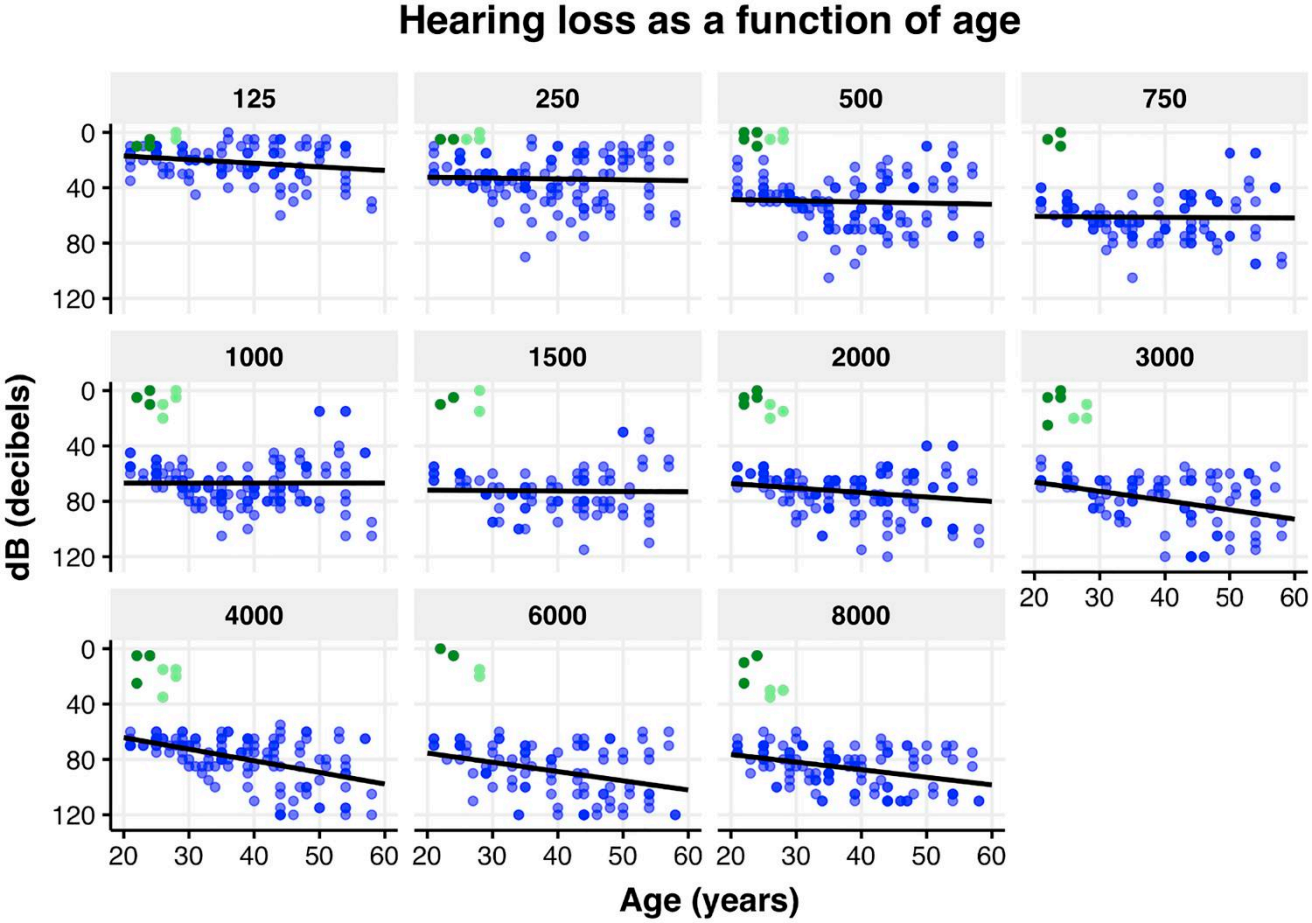
Hearing loss as function of age for each frequency for all USH2a (blue) and nsRP (green) patients.

**Figure 3:**
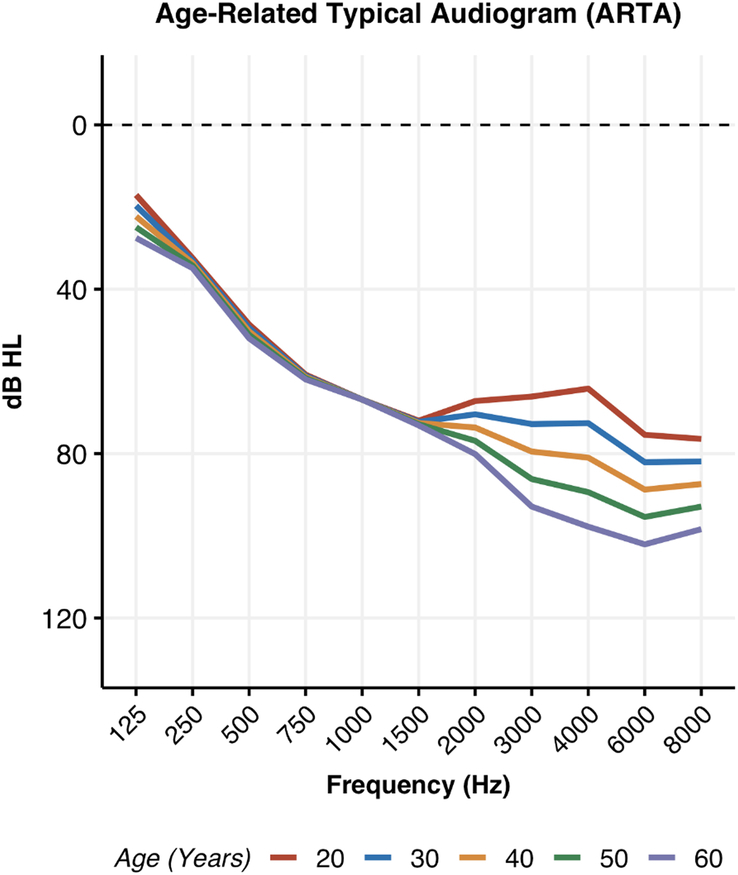
Age-related typical audiograms (ARTA) for all USH2a patients.

**Figure 4:**
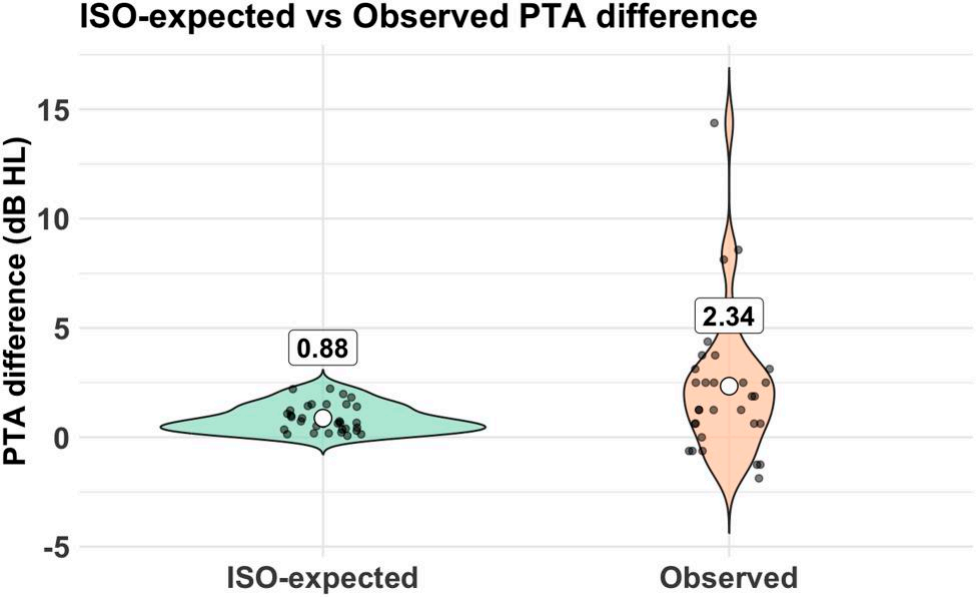
Comparison ISO-expected versus observed PTA_0.5-4kHz_ differences.

To evaluate the progression of hearing loss across frequencies, a regression model was created for each frequency to represent hearing loss as a function of age. Figure 2 shows the individual measurements plotted against a trend line, again showing the greatest deterioration in the higher frequencies. On the basis of these results, ARTAs were generated by predicting thresholds for specific ages (20, 30, 40, 50, and 60 y) at each frequency (Fig. 3). The observed progression in hearing loss between the 2 visits was compared against the expected hearing loss due to presbycusis, calculated based on age- and gender-matched data from the ISO 7029:2017 standard. This comparison, assessed using a Wilcoxon signed-rank test, showed that the average PTA_0.5-4kHz_ progression of 2.3 dB hearing loss in our cohort was significantly greater than the 0.9 dB HL expected from presbycusis (*P*=0.012, Fig. 4). Significant differences were observed at the frequencies of 2, 4, and 8 kHz, indicating that the hearing deterioration in our cohort exceeds typical age-related decline.

To model the phoneme scores, a sigmoid function was defined, and a fitting process was applied for each combination of participant, visit, and ear. The R² value was calculated to assess the goodness of fit, with a mean R² of 0.97. The fitting process was successful in more than 93% of cases allowing SRTs to be extracted. Mixed model analysis showed no significant difference in SRT between visits, with an estimated effect of −0.92 dB over the follow-up period (*P*=0.29).

On the basis of the mean PTA progression, a power analysis could be performed for a placebo-controlled, blinded study with a paired design for a potential future therapeutic trial. A power of 0.8 and a study duration of 2 years would require 64 patients, while a study duration of 3 years would require 30 patients.

### Vestibular findings

Vestibular assessment was performed in a total of 35 patients. In one of the USH2a patients, VEMP measurements could not be performed due to a temporary malfunction of the Eclipse system, and in another patient with USH2a, caloric stimulation was not performed due to a recent ear infection. Table 2 summarizes the main results by medical diagnosis.

**Table 2:** Key findings vestibular evaluation by clinical diagnosis

Characteristic	USH2a (N=33)	nsRP (N=2)^a^
Velocity step test		
Normal, N (%)	30 (91)	2 (100)
Hypofunction, N (%)	3 (9)	–
Max velocity, ◦/s, median (IQR)	49 (38-67)	47 and 70
T constant, s, median (IQR)	17.1 (14.4-23.7)	17.4 and 25.1
Gesamt-amplitude, ◦, median (IQR)	888 (633-1211)	1171 and 1182
Caloric reflex test		
Normal, N (%)^b^	30 (94)	2 (100%)
Unilateral hypofunction, N (%)^b^	2 (6)	–
Bilateral hypofunction, N (%)^b^	–	–
Max velocity, ◦/s, median (IQR)	20 (15-27)	12 and 37.5
vHIT, N (%)		
Normal	31 (94)	2 (100%)
Unilateral hypofunction	–	–
Bilateral hypofunction	2 (6)	–
cVEMP, N (%)^c^		
Normal	21 (66)	2 (100%)
Unilateral hypofunction	6 (19)	–
Bilateral hypofunction	5 (16)	–
oVEMP, N (%)^c^		
Normal	8 (25)	1 (50%)
Unilateral hypofunction	6 (19)	–
Bilateral hypofunction	18 (56)	1 (50%)

^a^ The 2 exact values of the 2 nsRP patients are given instead of the median.

^b^ One of the USH2a patients did not complete the caloric reflex test due to recent ear infections.

^c^ One of the USH2a patients did not complete the cVEMP and oVEMP tests due to machine malfunction.

The velocity step test indicated normal vestibular function in 91% of the USH2a patients and in both nsRP patients. Three of the USH2a patients (9%) showed hypofunction on this test. Caloric reflex testing was abnormal in 2 of the USH2a patients (6%), and vHIT also showed bilateral posterior canal hypofunction in 2 USH2a patients, while all other measurements indicated normal vestibular function.

VEMP measurements showed a significantly higher percentage of abnormalities. In cVEMP measurements, hypofunction was found in 11 USH2a patients (34%), with unilateral abnormalities in 6 patients and bilateral abnormalities in 5 patients. No abnormal cVEMP measurements were found in the 2 nsRP patients. oVEMP measurements were outside the normal range in 24 USH2a patients (75%), with 6 patients showing unilateral hypofunction and 18 patients showing bilateral hypofunction. Out of the 11 patients showing abnormal cVEMP measurements, 10 also exhibited abnormal oVEMP responses. Bilateral hypofunction was also observed in one of the 2 nsRP patients. No significant difference was observed between truncating and nontruncating groups in the proportion of abnormal VEMP measurements (*P*=0.64).

### Questionnaires

Patient-reported outcomes were assessed using questionnaires administered at baseline and at final visit 4 years later. Scores on the Usher Lifestyle Survey, PHQ-9, and SF-12 showed no significant decline over the study period. At baseline, patients had a mean score of 61.6 on the SF-12, which assesses general well-being and quality of life on a 0–100 scale, with higher scores indicating better quality of life. Interestingly, the SF-12 score exceeded the assumed population average of 50±10, suggesting a higher perceived quality of life in our cohort.^23^ The mean PHQ-9 score was 5.0 out of 27, with higher scores indicating more severe depressive symptoms. This baseline score was slightly higher than the general population average (men: 3.1±3.5, women: 2.7±3.5).^24^

The SSQ questionnaire, which assesses hearing impairment across multiple domains on a scale of 0-10, where lower scores correspond to greater perceived difficulty, showed an average baseline total score of 5.7. For comparison, a middle-aged cohort with normal hearing reported a mean score of 7.8 (SD 1.2).^25^ The greatest difficulties were observed in spatial hearing (4.8), followed by speech perception (6.0) and quality of hearing (6.2). At the end of the follow-up period, a small but significant decline was observed in the overall score (5.2, *P*=0.01), as well as in the subdomains of speech perception (5.3, *P*=0.01) and quality of hearing (5.7, *P*=0.03).

Balance complaints were assessed using the DHI questionnaire. At baseline, the mean score was 10.3, with a score of 16 or higher considered indicative of mild disability. No significant progression in the total DHI score was observed during the study period.

## Discussion

The aim of this study was to characterize the vestibular phenotype of USH2a and to assess the progression of hearing loss using prospectively collected data. To the best of our knowledge, this is the largest study to date using prospectively collected data to analyze the rate of hearing loss progression in USH2a patients. Our results provide robust evidence of measurable hearing loss progression in PTA_0.5-4kHz_ thresholds in USH2a patients that exceeds typical presbycusis. Vestibular evaluation did not reveal any dysfunction of the vestibulo-ocular reflex obtained from the semicircular canals, although VEMP results suggest a potential subclinical impairment of the otolith organs in USH2a patients.

The mean audiograms of USH2a patients in our study showed symmetric bilateral high-frequency SNHL, consistent with earlier reports.^26,27^ The ATD of 0.6 dB/year in our study is in line with the cross-sectional data of Hartel et al.^27^, who reported an ATD of 0.4 to 0.5 dB/year at 0.25 to 2 kHz and 0.7 to 0.8 dB/year at 4 to 8 kHz in a retrospective cohort of 110 patients. The pattern of progression was also similar, with the most pronounced progression in the mid-to-high frequencies, being reflected by an ATD of 0 to 0.6 dB/year at 0.125 to 2 kHz and 1.0 to 1.6 dB/year at 4 to 8 kHz. In contrast, the RUSH2A study found no significant deterioration in PTA with increasing age, which may be due to the high interpatient variability in USH2a, highlighting the need for a larger cohort in cross-sectional analyses.^28^ The audiograms of the 2 nsRP patients were within normal hearing ranges, reflecting generally intact auditory function.

Although a progression in PTA was observed, phoneme score measured speech perception did not deteriorate during the follow-up period in the USH2a group. This might be due to the relatively moderate PTA decline during the 4-year follow-up (2.3 dB), which is unlikely to have a significant influence on phoneme-based speech recognition scores. Another contributing factor might be the relatively young mean age of our cohort (36.5 y), as patients with more severe visual symptoms were excluded. Pennings et al.^29^ reported in a previous study that speech performance began to decline at the age of 38 years at a rate of 0.4% per year. However, patient-reported outcomes from the SSQ questionnaire did show a small but significant decline in speech understanding. This discrepancy between stable SRT thresholds and worsening outcomes on the SSQ questionnaire could potentially be explained by compensatory strategies and increased listening effort and may be an indication that phoneme scores in a soundproof environment do not completely reflect the real-world speech difficulties experienced by patients.

The vestibular evaluation of our study showed that 2 (6%) of USH2a patients had hypofunction during caloric reflex testing, which is in line with previous findings of a 7.5% incidence in the general population.^30^ Furthermore, only 2 patients (6%) from our cohort had bilateral hypofunction during vHIT testing, which is generally consistent with the reported prevalence of 0% to 3.7% among healthy subjects.^30,31^ These normal vestibulo-ocular reflexes indicate an intact function of the semicircular canals in USH2a patients.

However, during VEMP testing, substantially higher rates of abnormal measurements were seen in our USH2a cohort. Abnormal oVEMP measurements were observed in 75% of patients and abnormal cVEMP measurements were seen in 34%. This is considerably higher than for the general population, where abnormal oVEMP and cVEMP results are reported in 3% to 22% and 1% to 7% of healthy control subjects, respectively.^32–34^ The increased incidence of abnormal VEMP results found in our study is consistent with previous studies among USH2a patients. For example, Magliulo et al.^14^ reported pathologic oVEMP results in 80% of a small series of 5 USH2a patients and bilateral absence of cVEMP in 40% of patients. Similarly, a study by Wafa et al.^15^ in a larger cohort found bilateral absence during cVEMP testing in 6 of 35 USH2a patients, representing 17% of the study population. This suggests that otolith organ function may be impaired in USH2a patients compared with the general population. However, this potentially reduced function does not appear to result in clinically significant balance complaints, as indicated by the low mean score of 10.3 on the DHI questionnaire. Previous research has also shown that objective vestibular test results and subjective patient-reported balance symptoms are poorly correlated and that patients with vestibular dysfunction do not always experience significant symptoms, leaving aside possible central compensatory mechanisms.^35,36^

Since only 2 nsRP patients were included in the vestibular analysis, the findings should be interpreted cautiously and cannot be generalized to the broader nsRP population. However, the finding of bilaterally absent cVEMP responses in one patient raises the possibility that otolith organ dysfunction may also be present in a subgroup nsRP patients, warranting further study in a larger cohort.

A major strength of this study is its prospective longitudinal design, which allowed direct assessment of hearing loss progression in individual patients over time. In addition, this study combined both objective audiological and vestibular test results with subjective patient-reported outcomes, providing a multidimensional perspective on functional impairment and its impact on everyday activities. Furthermore, combining both audiological and vestibular findings gives a more complete overview of the phenotypic profile. However, there are some limitations that need to be considered. The mean age in our cohort is relatively young, and therefore the results may not be directly generalizable to older USH2a patients, who may exhibit different rates of progression. Multiple audiologists conducted the measurements following a strict protocol to minimize variability, although some inter-rater variation may still have occurred. In addition, the vestibular examination was performed only once, so statements about possible progression of vestibular dysfunction cannot be made properly. Furthermore, the small number of nsRP patients restricts the ability to draw conclusions about this subgroup. Although the overall study population is relatively large compared with previous investigations in this field, it remains limited, necessitating cautious interpretation of our findings.

From a clinical point of view, the ATD found provides insight for the design of potential future clinical trials focusing on hearing preservation. For example, the progression of PTA could be used in a power analysis to determine an appropriate sample size for a placebo-controlled, blinded study. In addition, understanding typical progression rates can aid in clinical counseling, allowing health care providers to set realistic expectations regarding the natural course of hearing loss in USH2a. This information is also valuable in determining the optimal timing for auditory rehabilitation, such as the use of hearing aids or CIs.

Future research is needed with older and more diverse patient populations to improve generalizability and to determine whether the patterns observed in our cohort hold true across all age groups. In addition, prolonging the follow-up period or incorporating audioprofiling tests could help further capture clinically significant progression across different age groups. Longitudinal vestibular studies are also needed to determine whether otolith organ dysfunction is progressive and whether this leads to vestibular problems in later life. In addition, further exploration of genotype-phenotype correlations within *USH2A* variants may provide even more accurate predictions of disease severity and progression, which may help guide individualized patient management approaches.

In conclusion, this study shows strong longitudinal evidence of measurable hearing loss progression in PTA_0.5-4kHz_ thresholds in USH2a patients, particularly in the mid-to-high frequencies, whereas speech understanding, as measured by SRT, remains relatively stable. Vestibular assessment shows intact semicircular canal function, but VEMP testing revealed signs of otolith organ dysfunction, which did not appear to translate into significant clinical balance deficits. These results underscore the significance of comprehensive phenotypic assessment and provide a basis for future clinical trials and therapeutic interventions.

## Funding Sources

Funding for this study was provided by Stichting Ushersyndroom and the Radboud Fonds.

## Supplementary Material

## References

[1] Rosenberg T Haim M Hauch AM Parving A . The prevalence of Usher syndrome and other retinal dystrophy-hearing impairment associations. Clin Genet 1997;51:314–321. 9212179 10.1111/j.1399-0004.1997.tb02480.x

[2] Spandau UH Rohrschneider K . Prevalence and geographical distribution of Usher syndrome in Germany. Graefes Arch Clin Exp Ophthalmol 2002;240:495–498. 12107518 10.1007/s00417-002-0485-8

[3] Boughman JA Vernon M Shaver KA . Usher syndrome: definition and estimate of prevalence from two high-risk populations. J Chronic Dis 1983;36:595–603. 6885960 10.1016/0021-9681(83)90147-9

[4] Delmaghani S El-Amraoui A . The genetic and phenotypic landscapes of Usher syndrome: from disease mechanisms to a new classification. Hum Genet 2022;141:709–735. 35353227 10.1007/s00439-022-02448-7 PMC9034986

[5] Eudy JD Weston MD Yao S . Mutation of a gene encoding a protein with extracellular matrix motifs in Usher syndrome type IIa. Science 1998;280:1753–1757. 9624053 10.1126/science.280.5370.1753

[6] Hope CI Bundey S Proops D Fielder AR . Usher syndrome in the city of Birmingham--prevalence and clinical classification. Br J Ophthalmol 1997;81:46–53. 9135408 10.1136/bjo.81.1.46 PMC1721995

[7] Toualbi L Toms M Moosajee M . USH2A-retinopathy: From genetics to therapeutics. Exp Eye Res 2020;201:108330. 33121974 10.1016/j.exer.2020.108330 PMC8417766

[8] Koenekoop R Arriaga M Trzupek KM Lentz J . Usher syndrome type II. In: Adam MP, Feldman J, Mirzaa GM, Pagon RA, Wallace SE, Amemiya A, eds. GeneReviews(®). Seattle (WA): University of Washington, Seattle Copyright © 1993-2025, University of Washington, Seattle. GeneReviews is a registered trademark of the University of Washington, Seattle. All rights reserved; 1993.

[9] Lee SY Joo K Oh J . Severe or profound sensorineural hearing loss caused by novel USH2A variants in korea: potential genotype-phenotype correlation. Clin Exp Otorhinolaryngol 2020;13:113–122. 31674169 10.21053/ceo.2019.00990 PMC7248602

[10] Markova TG Lalayants MR Alekseeva NN . Early audiological phenotype in patients with mutations in the USH2A gene. Int J Pediatr Otorhinolaryngol 2022;157:111140. 35452909 10.1016/j.ijporl.2022.111140

[11] Hartel BPP Huygen LHM Homans PL Goedegebure NC , and v.W. A., E. S, A.F., Klaver, C.C., van den Born, L.I., and Pennings, R.J. Evaluation of hearing in patients with USH2A-associated nonsyndromic retinitis pigmentosa. Unpublished data, personal communication.

[12] Hartel BP van Nierop JWI Huinck WJ . Cochlear implantation in patients with Usher syndrome type IIa increases performance and quality of life. Otol Neurotol 2017;38:e120–e127. 28498263 10.1097/MAO.0000000000001441

[13] Fehrmann MLA Lanting CP Haer-Wigman L . Long-term outcomes of cochlear implantation in Usher syndrome. Ear Hear 2024;45:1542–1553. 38987893 10.1097/AUD.0000000000001544 PMC11487040

[14] Magliulo G Iannella G Gagliardi S . Usher’s syndrome type II: a comparative study of genetic mutations and vestibular system evaluation. Otolaryngol Head Neck Surg 2017;157:853–860. 28653555 10.1177/0194599817715235

[15] Wafa TT Faridi R King KA . Vestibular phenotype-genotype correlation in a cohort of 90 patients with Usher syndrome. Clin Genet 2021;99:226–235. 33089500 10.1111/cge.13868 PMC7821283

[16] Pierrache LH Hartel BP van Wijk E . Visual prognosis in USH2A-associated retinitis pigmentosa is worse for patients with usher syndrome type IIa than for those with nonsyndromic retinitis pigmentosa. Ophthalmology 2016;123:1151–1160. 26927203 10.1016/j.ophtha.2016.01.021

[17] Reurink J Weisschuh N Garanto A . Whole genome sequencing for USH2A-associated disease reveals several pathogenic deep-intronic variants that are amenable to splice correction. HGG Adv 2023;4:100181. 36785559 10.1016/j.xhgg.2023.100181 PMC9918427

[18] Pharma T Study to Evaluate Ultevursen in Subjects with Retinitis Pigmentosa (RP) Due to Mutations in Exon 13 of the USH2A Gene (LUNA): ClinicalTrials.gov; 2024. Accessed April 7, 2025. https://clinicaltrials.gov/search?term=NCT06627179

[19] Duncan JL Liang W Maguire MG . Baseline visual field findings in the RUSH2A study: associated factors and correlation with other measures of disease severity. Am J Ophthalmol 2020;219:87–100. 32446738 10.1016/j.ajo.2020.05.024 PMC8596302

[20] Recommended procedure for pure-tone bone-conduction audiometry without masking using a manually operated instrument. British Society of Audiology--technical note. Br J Audiol 1985;19:281–282. 4074981 10.3109/03005368509078985

[21] Huygen PLM Pennings RJE Cremers CWRJ . Characterizing and distinguishing progressive phenotypes in nonsyndromic autosomal dominant hearing impairment. Audiol Med 2003;1:37–46.

[22] Standardization IOf . Acoustics—Statistical Distribution of Hearing Thresholds Related to Age and Gender. Geneva: International Organization for Standardization. 2017;2017:1–22.

[23] Gandek B Ware JE Aaronson NK . Cross-validation of item selection and scoring for the SF-12 Health Survey in nine countries: results from the IQOLA Project. International Quality of Life Assessment. J Clin Epidemiol 1998;51:1171–1178. 9817135 10.1016/s0895-4356(98)00109-7

[24] Kocalevent RD Hinz A Brähler E . Standardization of the depression screener patient health questionnaire (PHQ-9) in the general population. Gen Hosp Psychiatry 2013;35:551–555. 23664569 10.1016/j.genhosppsych.2013.04.006

[25] Demeester K Topsakal V Hendrickx JJ . Hearing disability measured by the speech, spatial, and qualities of hearing scale in clinically normal-hearing and hearing-impaired middle-aged persons, and disability screening by means of a reduced SSQ (the SSQ5). Ear Hear 2012;33:615–616. 22568994 10.1097/AUD.0b013e31824e0ba7

[26] Leijendeckers JM Pennings RJ Snik AF Bosman AJ Cremers CW . Audiometric characteristics of USH2a patients. Audiol Neurootol 2009;14:223–231. 19129697 10.1159/000189265

[27] Hartel BP Löfgren M Huygen PL . A combination of two truncating mutations in USH2A causes more severe and progressive hearing impairment in Usher syndrome type IIa. Hear Res 2016;339:60–68. 27318125 10.1016/j.heares.2016.06.008

[28] Iannaccone A Brewer CC Cheng P . Auditory and olfactory findings in patients with USH2A-related retinal degeneration-Findings at baseline from the rate of progression in USH2A-related retinal degeneration natural history study (RUSH2A). Am J Med Genet A 2021;185:3717–3727. 34331386 10.1002/ajmg.a.62437 PMC8717864

[29] Pennings RJ Huygen PL Weston MD . Pure tone hearing thresholds and speech recognition scores in Dutch patients carrying mutations in the USH2A gene. Otol Neurotol 2003;24:58–63. 12544030 10.1097/00129492-200301000-00013

[30] Oliveira LNR Oliveira CLA Lopes KC Ganança FF . Diagnostic assessment of patients with Meniere’s disease through caloric testing and the video-head-impulse test. Braz J Otorhinolaryngol 2021;87:428–433. 31870737 10.1016/j.bjorl.2019.10.008 PMC9422366

[31] Grill E Heuberger M Strobl R . Prevalence, determinants, and consequences of vestibular hypofunction. results from the KORA-FF4 survey. Front Neurol 2018;9:1076. 30581415 10.3389/fneur.2018.01076 PMC6293194

[32] Xu H Liang FY Chen L . Evaluation of the utricular and saccular function using oVEMPs and cVEMPs in BPPV patients. J Otolaryngol Head Neck Surg 2016;45:12. 26857819 10.1186/s40463-016-0125-7 PMC4746908

[33] Aydin C Önay Ö Tezcan E Aşkar Z Özdek A . Comparison of cervical and ocular vestibular-evoked myogenic potential responses between tone burst versus chirp stimulation. Eur Arch Otorhinolaryngol 2022;279:2339–2343. 34129084 10.1007/s00405-021-06936-w

[34] Shahnaz N David EA . Normal values for cervical and ocular vestibular-evoked myogenic potentials using EMG scaling: effect of body position and electrode montage. Acta Otolaryngol 2021;141:440–448. 33641604 10.1080/00016489.2021.1887517

[35] Jeon E-J Lim C-H Son E-J Jeong C-Y Lim JH Lee HJ . Associations between Dizziness Handicap Inventory scores and vestibular function tests: a cross-sectional survey. Res Vestib Sci 2024;23:156–164.

[36] Deveze A Bernard-Demanze L Xavier F Lavieille JP Elziere M . Vestibular compensation and vestibular rehabilitation. Current concepts and new trends. Neurophysiol Clin 2014;44:49–57. 24502905 10.1016/j.neucli.2013.10.138

