# Supplementary material for: From Sound to Stability: Lessons Learned From the CRUSH Study on Hearing Loss Progression and Vestibular Phenotype in Usher Syndrome Type 2A
Source: Otol Neurotol. 2026 Feb 23;47(4):549–55. doi: 10.1097/MAO.0000000000004851 (PMC12970546; doi:10.1097/MAO.0000000000004851)
Supplement: Supplementary file 1 [file mao-47-549-s001.docx]

**Appendix 1.** *CRUSH Eligibility Criteria*

| **Participant inclusion criteria** | **Participant exclusion criteria** |
| --- | --- |
| 1. Clinically diagnosed with rod-cone degeneration and biallelic (likely) pathogenic variants in *USH2A* 2. Willing and able to complete the informed consent process 3. Ability to return for all study visits over 48 months 4. Age ≥ 16 years at enrolment . | 1. (Likely) pathogenic variants in genes that cause autosomal dominant RP, X-linked RP, or presence of biallelic variants in autosomal recessive RP/retinal dystrophy genes other than Usher genes 2. Expected to enter experimental treatment trial at any time during this study 3. History of more than one year of cumulative treatment, at any time, with an agent associated with pigmentary retinopathy (including hydroxychloroquine, chloroquine, thioridazine, and deferoxamine) 4. The audiometric PTA(1-2-4kHz) for the best hearing ear should not exceed 75 dB HL. Patients with bilateral cochlear implants cannot participate in the study. 5. A planned, second, cochlear implantation during the study |
| **Ocular inclusion criteria** | **Ocular exclusion criteria** |
| 1. Clinical diagnosis of a rod-cone degeneration 2. Clear ocular media and adequate pupil dilation to permit good quality photographic imaging. 3. Ability to perform kinetic and static perimetry reliably 4. Baseline visual acuity ETDRS letter score of 54 or more (approximate Snellen equivalent 20/80 or better) 5. Stable fixation 6. Clinically determined (on Octopus 900 Pro) kinetic visual field III4e area 7,5°, or more in the study eye. | 1. Current vitreous haemorrhage 2. Current or any history of hematogenous retinal detachment 3. Current or any history of (e.g., prior to cataract or refractive surgery) spherical equivalent of the refractive error worse than -8 Dioptres of myopia 4. History of intraocular surgery (e.g., cataract surgery, vitrectomy, penetrating keratoplasty, or LASIK) within the last three months 5. Current or any history of confirmed diagnosis of glaucoma (e.g., based on glaucoma visual field, nerve changes, or glaucoma filtering surgery) 6. Current or any history of retinal vascular occlusion or proliferative diabetic retinopathy 7. Expected to have cataract removal surgery during the study 8. History or current evidence of ocular disease that, in the opinion of the investigator, may confound assessment of visual function 9. History of treatment for retinitis pigmentosa that could affect the progression of retinal degeneration (including participation in a clinical trial within the last year or a retained drug delivery device) |
