# Supplementary material for: From Sound to Stability: Lessons Learned From the CRUSH Study on Hearing Loss Progression and Vestibular Phenotype in Usher Syndrome Type 2A
Source: Otol Neurotol. 2026 Feb 23;47(4):549–55. doi: 10.1097/MAO.0000000000004851 (PMC12970546; doi:10.1097/MAO.0000000000004851)
Supplement: Supplementary file 2 [file mao-47-549-s002.docx]

**Appendix 2.** *CRUSH protocol: visit schedule and testing procedures*

| **Testing Procedures** | **Visit 1** Baseline | **Visit 4^a^** 36 months | **Visit 5** 48 months |
| --- | --- | --- | --- |
| **Patient Reported Outcome (questionnaires)^b^** | X |  | X |
| **Demographics and medical history** | X |  |  |
| **Adverse events** | X | X | X |
| **Audiometry** | | | |
| Pure tone- and speech audiometry | X |  | X |
| Digits-in-noise test (DIN) | X |  |  |
| Otoacoustic emissions (OAE) | X |  | X |
| **Vestibular exam** | | | |
| Velocity step test (VST) |  | X^c^ |  |
| Caloric reflex test |  | X^c^ |  |
| Video head impulse tests (vHIT) |  | X^c^ |  |
| Cervical vestibular evoked myogenic potentials (cVEMP) |  | X^c^ |  |
| Ocular vestibular evoked myogenic potentials (oVEMP) |  | X^c^ |  |

**^a^** At visit 2 and 3 only ophthalmic tests were performed.
^b^ Performed questionnaires are Speech, Spatial and Qualities of Hearing Scale (SSQ), Dizziness Handicap Inventory (DHI), Usher lifestyle survey, 12-item Short-from-Health Survey (SF-12), and Patient Health Questionnaire Mood Scale (PHQ-9).
^c^ Vestibular exam took place in the third or fourth year.

**Audiometry**

Pure-tone air and bone conduction thresholds for frequencies ranging from 0.25 to 8 kHz were assessed using standard clinical procedures in a sound-isolated environment. Bone conduction thresholds were measured to rule out conductive hearing loss.

Speech perception in quiet was assessed using monosyllabic CVC words presented from the front, following the method described by Bosman and Smoorenburg (1). Performance-intensity functions were obtained by recording the percentage of correctly identified phonemes at increasing presentation levels (40-120 dB HL). The speech recognition threshold (SRT), defined as the level at which 50% of phonemes are correctly recognized, was derived from these functions.

Speech perception in noise was assessed using the DIN test, a full-bandwidth digit-triplet test presented in long-term average speech spectrum (LTASS) noise. This test uses an adaptive one-up one-down procedure to determine the SRT, corresponding to the signal-to-noise ratio (SNR) required for 50% correct responses over whole triplets (2). The noise level was fixed at 65 dB, while the speech level started at 65 dB (SNR = 0), with subsequent adjustments made in 2 dB increments until the SRT was obtained.

DPOAEs were measured in a soundproof environment over a range of stimulus frequencies from 1000 to 8000 Hz. A Fast Fourier Transform (FFT) was used to analyze the response data and estimate DPOAE levels at the 2f1-f2 frequency. Noise levels at the DPOAE frequency were assessed by comparing the two response buffers, ensuring reliability and accuracy of the DPOAE measurements. DPOAE responses were considered absent if the DPOAE was not present at two or more f2 frequencies with at least 3-6 dB SNR.

**Vestibular evaluation**

Oculomotor tests were performed to rule out central lesions. Caloric reflex testing was performed with bithermal (30°C and 44°C) water irrigation of the external auditory canal for 30 seconds.

VST was performed with the patient seated in a rotating chair with the head anteflexed at 30°. Vestibular reactivity was evaluated for both directions (2^0^/s acceleration, maximum velocity 90^0^/s, -200^0^/s deceleration). The vHIT was performed with the vHIT Ulmer II system (Synapsys, France), recording eye movements at 100 Hz. Eye gain was calculated for the six semicircular canals by dividing eye velocity by head velocity. Cervical- and ocular-VEMP testing on a clinical Eclipse II system (Interacoustics, Denmark) used auditory stimuli to assess saccule and utricle function, with surface electrodes placed on the sternocleidomastoid muscle to obtain the inhibitory myogenic response (cVEMP) and around the eyes to record excitatory muscle response of the inferior oblique (oVEMP). Areflexia was defined as the absence of responses during vestibular testing, whereas hyporeflexia was defined by responses below normal thresholds. For the caloric reflex test, these thresholds are <7°/s for cold irrigation and <10°/s for warm irrigation. For the VST, the thresholds are a gain of <33%, a maximum slow-phase velocity of <30°/s and a time constant of <11 s. For the vHIT, the thresholds are a gain of <0.7 for the vertical semicircular canals and <0.8 for the horizontal semicircular canals and for the VEMP an absence of reactivity below 100 dB HL.

**References**

1. Bosman AJ, Smoorenburg GF. Intelligibility of Dutch CVC syllables and sentences for listeners with normal hearing and with three types of hearing impairment. Audiology. 1995;34(5):260-84.

2. Smits C, Theo Goverts S, Festen JM. The digits-in-noise test: assessing auditory speech recognition abilities in noise. J Acoust Soc Am. 2013;133(3):1693-706.
