# Supplementary material for: From Sound to Stability: Lessons Learned From the CRUSH Study on Hearing Loss Progression and Vestibular Phenotype in Usher Syndrome Type 2A
Source: Otol Neurotol. 2026 Feb 23;47(4):549–55. doi: 10.1097/MAO.0000000000004851 (PMC12970546; doi:10.1097/MAO.0000000000004851)
Supplement: Supplementary file 3 [file mao-47-549-s003.docx]

**Appendix 3**. *Enrolment flowchart of the CRUSH study*

USH2a

33 patients

41 patients screened

4 patients did not meet inclusion criteria due to limited visual field

37 patients included

35 patients

2 patients withdrawn from study

nsRP

2 patients
