# Supplementary material for: From Sound to Stability: Lessons Learned From the CRUSH Study on Hearing Loss Progression and Vestibular Phenotype in Usher Syndrome Type 2A
Source: Otol Neurotol. 2026 Feb 23;47(4):549–55. doi: 10.1097/MAO.0000000000004851 (PMC12970546; doi:10.1097/MAO.0000000000004851)
Supplement: Supplementary file 4 [file mao-47-549-s004.docx]

**Appendix 4.** *Audiological baseline characteristics by clinical diagnosis*

| **Characteristic** | **USH2a**  **(N = 32)^a^** | **nsRP**  **(N = 2)^b^** |
| --- | --- | --- |
| **PTA_0.5-4kHz_, dB HL** |  |  |
| Mean (SD) | 66.1 (14.3) | 1.3 and 7.5 |
|  |  |  |
| **SRT, dB** |  |  |
| Mean (SD) | 78.2 (11.6) | 21.7 and 32.0 |
|  |  |  |
| **SRT DIN, dB** |  |  |
| Mean (SD) | 4.2 (3.4) | -8.4 and -6.0 |
|  |  |  |
| **DPOAE** |  |  |
| Present | 7 (22%) | 2 (100%) |
| Absent | 24 (75%) | - |
| Missing | 1 (3%) | - |
|  |  |  |
| **Use of hearing aid** | 29 (91%) | - |
|  |  |  |
| **Use of cochlear implant** | - | - |
|  |  |  |
| **History of ear infections** | 15 (47%) | 1 (50%) |
|  |  |  |
| **History of head injury/meningitis** | 6 (19%) | - |
|  |  |  |
| **Tinnitus** | 10 (31%) | - |
|  |  |  |
| **Exposed to heavy noise in the past** | 7 (22%) | 1 (50%) |
|  |  |  |
| **Long term use of any antibiotics** | 6 (19%) | - |
|  |  |  |

**^a^** The two exact values of the two nsRP patients are given instead of the mean
