# Supplementary material for: From Sound to Stability: Lessons Learned From the CRUSH Study on Hearing Loss Progression and Vestibular Phenotype in Usher Syndrome Type 2A
Source: Otol Neurotol. 2026 Feb 23;47(4):549–55. doi: 10.1097/MAO.0000000000004851 (PMC12970546; doi:10.1097/MAO.0000000000004851)
Supplement: Supplementary file 5 [file mao-47-549-s005.docx]

**Appendix 5**. *Genotype and clinical diagnosis summary*

| **Study number** | **c.DNA** | **pNomen** | **Coding effect** | **c.DNA** | **pNomen** | **Coding effect** | **Clinical Diagnosis** |
| --- | --- | --- | --- | --- | --- | --- | --- |
| 1. | c.2299del | p.(Glu767Serfs*21) | Frameshift | c.1227G>A | p.(Trp409*) | Nonsense | USH2a |
| 2. | c.2299del | p.(Glu767Serfs*21) | Frameshift | c.1227G>A | p.(Trp409*) | Nonsense | USH2a |
| 3. | c.2299del | p.(Glu767Serfs*21) | Frameshift | c.9959-2A>G | p.? | Possible splice effect | USH2a |
| 4. | c.2299del | p.(Glu767Serfs*21) | Frameshift | c.9959-2A>G | p.? | Possible splice effect | USH2a |
| 5. | c.2299del | p.(Glu767Serfs*21) | Frameshift | c.949C>A | p.(Arg317=) | Synonymous | USH2a |
| 6. | c.2299del | p.(Glu767Serfs*21) | Frameshift | c.1606T>C | p.(Cys536Arg) | Missense | USH2a |
| 7. | c.2299del | p.(Glu767Serfs*21) | Frameshift | c.2276G>T | p.(Cys759Phe) | Missense | USH2a |
| 8. | c.2299del | p.(Glu767Serfs*21) | Frameshift | c.1434G>C | p.(Glu478Asp) | Missense | USH2a |
| 9. | c.2299del | p.(Glu767Serfs*21) | Frameshift | c.14803C>T | p.(Arg4935*) | Nonsense | USH2a |
| 10. | c.2299del | p.(Glu767Serfs*21) | Frameshift | c.15053-2A>T | p.? | Possible splice effect | USH2a |
| 11. | c.2299del | p.(Glu767Serfs*21) | Frameshift | c.7187G>A | (p.Trp2396*) | Nonsense | USH2a |
| 12. | c.2299del | p.(Glu767Serfs*21) | Frameshift | c.7595-2144A>G | p.? | Possible splice effect | USH2a |
| 13. | c.2299del | p.(Glu767Serfs*21) | Frameshift | c.7950dup | p.(Asn2651Glnfs*10) | Frameshift | USH2a |
| 14. | c.2299del | p.(Glu767Serfs*21) | Frameshift | c.9390G>A | p.(Tip3130*) | Nonsense | USH2a |
| 15. | c.2299del | p.(Glu767Serfs*21) | Frameshift | c.9842G>T | p.(Cys3281Phe) | Missense | USH2a |
| 16. | c.949C>A | p.(Arg317=) | Synonymous | c.11676del | p.(Lys3892Asnfs*41) | Frameshift | USH2a |
| 17. | c.949C>A | p.(Arg317=) | Synonymous | c.11676del | p.(Lys3892Asnfs*41) | Frameshift | USH2a |
| 18. | c.949C>A | p.(Arg317=) | Synonymous | c.949C>A | p.(Arg317=) | Synonymous | USH2a |
| 19. | c.949C>A | p.(Arg317=) | Synonymous | c.920_923dup | p.(His308Glnfs*16) | Frameshift | USH2a |
| 20. | c.949C>A | p.(Arg317=) | Synonymous | c.4773del | p.(Val1592*) | Nonsense | USH2a |
| 21. | c.1256G>T | p.(Cys419Phe) | Missense | c.1606T>C | p.(Cys536Arg) | Missense | USH2a |
| 22. | c.1256G>T | p.(Cys419Phe) | Missense | c.11864G>A | p.(Trp3955*) | Nonsense | USH2a |
| 23. | c.1256G>T | p.(Cys419Phe) | Missense | c.(785-?_+5572+?)dup | p.? | Large duplication | USH2a |
| 24. | c.5516T>A | p.(Val1839Glu) | Missense | c.2081G>A | p.(Cys694Tyr) | Missense | USH2a |
| 25. | c.5516T>A | p.(Val1839Glu) | Missense | c.5018T>C | p.(Leu1673Pro) | Missense | USH2a |
| 26. | c.920_923dup | p.(His308Glnfs*16) | Frameshift | c.920_923dup | p.(His308Glnfs*16) | Frameshift | USH2a |
| 27. | c.920_923dup | p.(His308Glnfs*16) | Frameshift | c.920_923dup | p.(His308Glnfs*16) | Frameshift | USH2a |
| 28. | c.1036A>C | p.(Ans346His) | Missense | c.(7301-?_10939+?)del | p.? | Large deletion | USH2a |
| 29. | c.10525A>T | p.(Lys3509*) | Nonsense | c.10525A>T | p.(Lys3509*) | Nonsense | USH2a |
| 30. | c.1606T>C | p.(Cys536Arg) | Missense | c.5516T>A | p.(Val1839Glu) | Missense | USH2a |
| 31. | c.1808G>A | p.(Gly603Glu) | Missense | c.7501C>T | p.(Gln2501*) | Nonsense | USH2a |
| 32. | c.1876C>T | p.(Arg626*) | Nonsense | c.14131C>T | p.(Gln4711*) | Nonsense | USH2a |
| 33. | c.8079G>A | p.(Trp2693*) | Nonsense | c.12806C>A | p.(Pro4269His) | Missense | USH2a |
| 34. | c.12343C>T | p.(Arg4115Cys) | Missense | c.13274C>T | p.(Thr4425Met) | Missense | nsRP |
| 35. | c.2276G>T | p.(Cys759Phe) | Missense | c.4697A>G | p.(Gln1566Arg) | Missense | nsRP |
